# Hospital Malnutrition in Latin America: Building a Culture of Nutrition Care: The feedM.E. Global Study Group Response to “A Quick Fix for Hospital-Acquired Malnutrition?”

**DOI:** 10.1177/0148607115592674

**Published:** 2016-04-26

**Authors:** Maria Isabel Correia

In Latin American hospitals, the malnutrition numbers are strikingly high; disease-related malnutrition has been reported in nearly 50% of adult patients in Argentina, Brazil, Chile, Costa Rica, Cuba, Dominican Republic, Ecuador, Mexico, Panama, Paraguay, Peru, Puerto Rico, Venezuela, and Uruguay.^1–6^

To address this problem, we worked as part of a feedM.E. Latin American Study Group. Together, we gathered evidence of how malnutrition incurs excessive human and financial tolls on healthcare systems in Latin America and how appropriate nutrition care can improve patients’ clinical outcomes and reduce healthcare costs.^7^ Despite abundant evidence, we found that hospital malnutrition is often overlooked and undertreated.^1,8,9^ Clinicians do not consistently follow best nutrition practices because barriers—lack of awareness, time, money, and training—stand in the way.^10–12^

Our study was intended as a call to action for healthcare professionals throughout Latin America, suggesting use of a simple and efficient Nutrition Care Pathway to screen all patients on admission or at initiation of care, assess their needs and provide supportive nutrition when needed, and deliver routine follow-up care with postdischarge nutrition planning, treatment, and monitoring.

In an invited commentary for the *Journal of Parenteral and Enteral Nutrition*, Juan B. Ochoa Gautier^13^ noted that lack of caregiver awareness was not the only cause of hospital-acquired malnutrition. Ochoa Gautier expressed concern that the problem was also related to limited understanding of the etiology of disease-related malnutrition, as well as shortfalls in identifying and addressing other barriers to delivery of optimal nutrition care specific to each hospital.

Surely most healthcare professionals in Latin America and elsewhere would agree with Ochoa Gautier that best-practice nutrition care happens in hospital and community health systems when

- Interdisciplinary nutrition care teams are widely available, and team members have an up-to-date understanding of malnutrition, including its etiology, diagnosis, and treatment.
- Nutrition screening, assessment, and treatment protocols are in place and routinely used to guide local practices.
- Local financial, social, and educational barriers to best-practice nutrition care are continuously identified and tirelessly addressed though quality improvement programs.

We fully appreciate the best-case scenario that Ochoa Gautier is championing for nutrition care in critically ill patients. We agree that these strategies represent great standards to guide hypothesis testing and quality improvement investigations toward optimal nutrition care. However, we feel that perfect nutrition therapy is hard to pinpoint in real-life situations, so we encourage simple initiatives to tackle malnutrition as a “big-picture” problem.

With the background described in our recent article, we proposed a Nutrition Care Pathway as a simple starter step to help build a hospital culture that includes attention to nutrition screening and offers prompt nutrition care when it is needed.^7^ People who are hospitalized often have preexisting nutrition compromise. In addition, our underlying premise is that hospitalized patients are at increased risk of malnutrition by way of disease-related inflammatory processes that elevate nutrient needs and lower appetite/intake. The sooner the disease-related nutrition gap is addressed, the more likely the patient is to recover faster and better. Major medical and nutrition societies recommend prompt nutrition intervention for patients who are hospitalized and malnourished.^14,15^

Our study aims to attract attention to the consequences of hospital malnutrition and to inspire incremental improvement of nutrition care. We feel these aims are especially important in Latin American countries, where high malnutrition rates persist among hospitalized patients.

That said, there are no “bad” ways to address malnutrition in Latin America or anywhere else in the world. We applaud Ochoa Gautier for his commitment to research on the pathophysiology of disease-related malnutrition and for his support of improving nutrition care in hospitals.

Maria Isabel Correia, MD, PhD, for the feedM.E. Global Study Group *Universidade Federal de Minas Gerais, Brazil*
